# Relevant and selective activity of *Pancratium illyricum* L. against *Candida albicans* clinical isolates: a combined effect on yeast growth and virulence

**DOI:** 10.1186/1472-6882-14-409

**Published:** 2014-10-23

**Authors:** Francesca Bonvicini, Fabiana Antognoni, Carmelina Iannello, Andrea Maxia, Ferruccio Poli, Giovanna Angela Gentilomi

**Affiliations:** Department of Pharmacy and Biotechnology, University of Bologna, via Massarenti 9, 40138 Bologna, Italy; Department for Life Quality Studies, University of Bologna, Corso Augusto, 237, 47921 Rimini, Italy; Department of Pharmacy and Biotechnology, University of Bologna, via Irnerio 42, 40126 Bologna, Italy; Interacademic Union for Studying of Secondary Metabolites of Vegetal Origin (Co.S.Me.Se.), Viale Ignazio da Laconi 13, Cagliari, Italy

**Keywords:** *Candida albicans* clinical isolates, Plant-derived compounds, Antifungal activity, Collagenase activity, *Pancratium illyricum* L

## Abstract

**Background:**

Alkaloids present in plants of the Amaryllidaceae family are secondary metabolites of high biological interest, possessing a wide range of pharmacological activities. In the search for new plant-derived compounds with antimicrobial activities, two alkaloid extracts obtained from bulbs and leaves of *Pancratium illyricum* L., a plant of the Amarillydaceae family, were tested for their effect on bacterial and yeast growth.

**Methods:**

The broth microdilution susceptibility test was applied to study the effect of plant extracts on the growth of reference bacterial strains and *Candida albicans* reference and clinical isolates strains. Extracts obtained from the different parts of the plant were tested and compared with the pure components identified in the extracts. Since matrix metalloproteinase enzymes play a role in the dissemination process of *Candida albicans*, the effect of the bulb extract and pure alkaloids on *in vitro* collagenase activity was tested. Cell viability test was carried out on human embryo lung fibroblasts (HEL 299).

**Results:**

Whilst both extracts did not show any inhibitory activity against neither Gram positive nor Gram negative bacteria, a strong antifungal activity was detected, in particular for the bulb extract. All clinical isolates were susceptible to the growth inhibitory activity of the bulb extract, with endpoint IC_50_ values ranging from 1.22 to 78 μg/mL. The pure alkaloids lycorine and vittatine, identified as components of the extract, were also assayed for their capacity of inhibiting the yeast growth, and lycorine turned very active, with endpoint IC_50_ values ranging from 0.89 to 28.5 μg/mL. A potent inhibition of the *in vitro* collagenase activity was found in the presence of the bulb extract, and this effect was much higher than that exerted by the pure alkaloids. Viability of cell lines tested was not affected by the extract.

**Conclusions:**

Taken together, results suggest that the extract of *Pancratium illyricum* may act as antifungal agent both directly on the yeast growth and by altering the tissue invasion process.

**Electronic supplementary material:**

The online version of this article (doi:10.1186/1472-6882-14-409) contains supplementary material, which is available to authorized users.

## Background

*Candida albicans* is an important pathogen of humans characterized by a high versatility. The yeast is a commensal in several anatomically distinct sites and, under an extensive variety of predisposing factors (ranging from specific immune defects to mucosal or cutaneous barrier disruption, ageing, diabetes, AIDS), it can invade tissues leading to a wide spectrum of clinical symptoms.

Among the early events in the pathogenesis of candidiasis there are the adhesion of the yeast cells to host cells, the yeast-hyphal transition, with changes in antigen expression and tissue affinities, and the direct persorption into the submucosa [1]. Subsequently, for the dissemination, *C. albicans* can affect host matrix metalloproteinases (MMPs) expression, mainly MMP-9, leading to tissue degradation and inflammatory processes [2].

MMPs are a family of secreted or cell surface-associated proteolytic enzymes targeting almost all extracellular matrix (ECM) components and basement membrane proteins [3]. MMPs have been classified according to their substrate specificities and are often referred to as collagenases, gelatinases, stromelysins, matrilysins, and membrane-type MMPs. The catalytic domain of all MMPs contains a zinc-binding motif, an additional structural zinc ion, and 2–3 calcium ions, which are required for the stability and the expression of enzyme activity [4]. To date, many extracts or compounds from plant origin have been studied as possible inhibitors of some MMPs as the modulation of their expression has been implicated in inflammatory disorders, cancer invasion, metastasis, and microbial infections [5, 6].

Concerning antifungal therapy, candidiasis are widely treated with triazole drugs (fluconazole, voriconazole, itraconazole, and posaconazole) that inhibit the biosynthesis of the ergosterol, the major sterol in the fungal plasma membrane. However, their extensive use has raised concern about resistant infections that negatively impact the clinical outcome mainly for high risk patients and those with persistent mycoses [7].

In the search for naturally derived compounds with promising antimicrobial activities, a particular focus was recently given to the alkaloids produced by plants of the *Amaryllidaceae* family. Indeed, the *Amaryllidaceae* alkaloids are secondary metabolites of high biological interest, possessing, among others, antimicrobial, antitumoral and acetylcholinesterase inhibitory activities [8–10]. Several species of *Crinum* have already been proven for their antibacterial and antifungal activities. For instance, the alkaloid crinamine isolated from bulbs of *C. jagus* showed strong inhibitory activity against *Staphylococcus aureus* [11], and *in vitro* activity against *C. albicans* was demonstrated for *C. macowanii* extract [12]. Alkaloids present in *C. angustum* Steud [13] and *C. purpurascens* [14] were also reported to possess antibacterial and antifungal activities.

In the present study, a set of 30 *C. albicans* clinical isolates was tested to explore the antifungal activity of an alkaloid extract of *Pancratium illyricum* L.*,* a species endemic to Sardinia, Corsica and the Tuscan archipelago [15] *;* the *in vitro* anti-collagenase activity and the mechanism of action of *P. illyricum* extract towards the collagenase enzyme were also investigated.

This work was aimed at finding new antifungal agents having anti-collagenase activity, which may also affect the yeast virulence, in a combined effect with growth inhibition.

## Methods

### Clinical isolates

The study included 30 *C. albicans* clinical isolates recovered from urine samples, collected at S. Orsola-Malpighi Hospital, Bologna (Italy). All isolates were cultured on CHROMagar Orientation Medium (Becton Dickinson, Heidelberg, Germany), then species identification was carried out by standard procedures, including colony morphology on chromogenic agar (CHROMagar Candida Medium, Becton Dickinson), and confirmed by MALDI Biotyper System using matrix-assisted laser desorption ionization–time of flight mass spectrometry (MALDI-TOF MS, Bruker Daltonik, GmbH, Germany) [16].

### Plant material

*Pancratium illyricum* L. was collected during the flowering period (May 2011) in the South of Sardinia (Punta San Michele, CA, Italy), and identified by Professor Mauro Ballero (University of Cagliari, Italy). A voucher specimen (CAG 1365) has been deposited in the Institute of Botany, University of Cagliari.

### Extraction and isolation of pure alkaloids

An extract enriched in alkaloids was prepared from bulbs and leaves separately. Fresh plant material (298 g and 168 g, respectively) was crushed in small pieces and exhaustively extracted with MeOH at room temperature for 72 h. The extracts were evaporated under reduced pressure to yield 15.85 g and 10.17 g for bulbs and leaves, respectively. These crude extracts were acidified by dissolving in 100 mL H_2_SO_4_ 1% (v/v) and neutral material was removed with *n*-hexane (6 × 100 mL) and chloroform (CHCl_3_, 4 × 100 mL). The acidified solution was then basified with 25% NH_4_OH up to pH 9–10 and extracted with CHCl_3_ (4 × 100 mL) to give the CHCl_3_ extract containing alkaloids (130 mg for bulbs and 120 mg for leaves). Both extracts were dried with anhydrous Na_2_SO_4_, filtered and completely dried under reduced pressure. The extract yields, on a fresh weight basis, were 0.05% for bulbs and 0.07% for leaves. The CHCl_3_ bulb extract was then subjected to vacuum liquid chromatography (VLC) [13] using a silica gel 60 Å (6–35 μm) column (1 cm diameter and 4 cm height). Alkaloids were eluted using *n*-hexane gradually enriched with ethylacetate (EtOAc), then gradually enriched with CHCl_3_ and finally with a mixture of EtOAc and CHCl_3_ gradually enriched with MeOH. Fractions of 10 mL were collected (200 in total), monitored by TLC (Dragendorff’s reagent, UV 254 nm) and combined according to their profiles. Nine main fractions were obtained and subjected to preparative TLC (20 cm × 20 cm × 0.25 mm, silica gel 60 F254). Crystals of lycorine and vittatine soluble compounds were obtained in major quantities from fractions *128*–*133* (eluted from VLC with *n*-Hexane-EtOAc-CHCl_3_, 20:60:20 to 15:60:25). Crystals of lycorine (20 mg) were collected as needles by separating from the solvent and then subjected to GC-MS analysis to confirm the alkaloid identity. Vittatine (7 mg) was obtained through preparative TLC from collected fractions (EtOAc-CHCl_3_-CH_3_OH 3:1:1 + 25% ammonia).

EIMS were obtained on a GC-MS Hewlett-Packard 6890 + MSD 5975 operating in EI mode at 70 eV. A HP-5 MS column (30 m × 0.25 mm × 0.25 μm) was used. The temperature program was: 100–180°C at 15°C min-1, 1 min hold at 180°C, 180–300°C at 5°C min-1 and 1 min hold at 300°C. Injector temperature was 280°C. The flow rate of the carrier gas (helium) was 0.8 mL min-1.

The compounds of the final CHCl_3_ extracts of bulbs and leaves, and the pure compounds lycorine and vittatine, were identified as TMS with the help of the NIST 05 database (NIST Mass Spectral Database, PC-Version 5.0, 2005, National Institute of Standardisation and Technology, Gaithersburg, MD, USA), and other plant-specific databases: the Golm Metabolome Database ( http://csbdb.mpimp-golm.mpg.de/csbdb/gmd/home/gmd_sm.html), on the basis of matching mass spectra and Kovats retention indexes (RI). The measured mass spectra were deconvoluted by the Automated Mass Spectral Deconvolution and Identification System (AMDIS 2.64, NIST Gaithersburg, MD) before comparison with the databases. RI values of the compounds were measured with standard n-hydrocarbon calibration mixture (C9–C36; Restek, Bellefonte, PA, USA, catalogue no. 31614, supplied by Teknokroma, Spain) using AMDIS 2.64 software. Additional files 1, 2, 3, 4, 5, 6 and 7 show the GC-MS profile of the bulb extract and pure compounds identified.

### Susceptibility testing

Preliminary experiments were performed on standard American Type Culture Collection (ATCC) strains: *Staphylococcus aureus* ATCC 25923, *Staphylococcus epidermidis* ATCC 12228, *Escherichia coli* ATCC 25922, *Klebsiella pneumoniae* ATCC 9591, and on *C. albicans* ATCC 10231.

Bacteria strains were assayed with the alkaloid extracts of *P. illyricum*, from bulbs and leaves (12 dilutions; range 0.156 to 312 μg/mL), and gentamicin (Sigma-Aldrich, Saint Louis, USA) as reference drug control, by means of a previously described broth microdilution protocol [13].

All yeast strains were assayed with the alkaloid extracts of *P. illyricum*, from bulbs and leaves, (12 dilutions; range range 0.156 to 312 μg/mL), the pure compounds lycorine and vittatine (12 dilutions; range 0.03 to 114 μg/mL and 0.05 to 225 μg/mL, respectively) and amphotericin B (12 dilutions; range 0.002 to 5 μg/mL). Amphotericin B was supplied from Sigma (Sigma-Aldrich, Saint Louis, USA).

Inocula of all *Candida* isolates and two-fold microdilution broth method were performed according to the European Committee on Antimicrobial Susceptibility Testing (EUCAST) guidelines [17]. Briefly, the inoculum was prepared by suspending a maximum of 5 distinct colonies in 5 mL of sterile 0.9% saline solution, then density was adjusted at 0.5 McFarland by measuring the absorbance (DU-530 UV–vis Spectrophotometer, Beckman Coulter, Inc., CA, USA). Finally, a working suspension was prepared from a 1:10 dilution of the standardized suspension in RPMI-1640 medium (Gibco^®^, Thermo Fisher Scientific Inc., Waltham, USA), containing glucose 2%, 0.3% levo-glutamine, 0.165 M 3-(N-morpholino)-propanesulfonic acid (MOPS), pH 7.0, to yield 1–5 × 10^5^ CFU/mL.

Each well of a 96-well microplate was inoculated with 100 μL of the yeast suspension and with 100 μL of the testing drugs, serially two-fold diluted in RPMI-1640 medium. Growth control wells containing 100 μL of sterile drug-free medium and 100 μL of the same inoculum were also prepared, as well as negative control wells. Finally, solvent controls were prepared by serially diluting DMSO (12 dilutions; starting from 3.12%) to measure its interfering effect on yeast growth. Each strain and all controls were tested in triplicate. The microdilution plates were incubated at 37°C for 24 h, without agitation, and subsequently the absorbance at 530 nm was measured by the Multiskan Ascent microplate reader (Thermo Fisher Scientific Inc., Waltham, USA). The growth inhibition of the yeast cells was calculated and expressed as MIC (minimum inhibitory concentration) and IC_50_ (concentration required for a growth reduction of the 50%).

The minimal fungicidal concentrations (MFCs) were determined by subculturing 50 μL of supernatants from the wells where no growth was observed in broth microdilution plates, previously described. The samples were cultured on Sabouraud agar plates (Becton Dickinson, Heidelberg, Germany) and incubated at 37°C for 24 h. The MFC was the lowest drugs concentration that showed either no growth or fewer than 3 colonies to obtain approximately 99 to 99.5% killing activity [18]. The MFC was determined from three independent experiments.

### Anti-collagenase assay

The bulbs extract of *P. illyricum* and pure compounds, lycorine and vittatine, were tested for their capacity of inhibiting *in vitro* collagenase activity. The assay employed was based on a spectrophotometric method reported in the literature [19], slightly modified for a 96-well microplate, using *Clostridium hystolyticum* collagenase (EC.3.4.23.3) and FALGPA (N-[3-(2-Furyl)acryloyl]-Leu-Gly-Pro-Ala) as substrate. Enzyme was dissolved in 50 mM Tricine buffer (containing 10 mM CaCl_2_ and 400 mM NaCl), pH 7.5, to give 0.8 units/mL (according to the supplier’s activity data); a 2 mM solution of substrate FALGPA was prepared in the same buffer. Twenty-five μL buffer, 25 μL H_2_O or inhibitor, and 25 μL enzyme were loaded into each well of the microtplate, and after 15 min preincubation, 50 μL of substrate were added. Absorbance was measured at 340 nm immediately and at 2-min intervals for 20 min. Enzyme activity was estimated by following the decrease in absorbance during the time interval due to substrate hydrolysis. Epigallocatechin gallate (EGCG) was used as positive control. Four substrate concentrations in the range 0.5 mM - 2.5 mM were assayed to elucidate the mechanism of inhibition.

### Cell viability testing

Cytotoxic effects of the bulbs extract of *P. illyricum* were evaluated against the human embryo lung fibroblasts (HEL 299) by using the commercially available alamarBlue Assay (Life Technologies, Thermo Fisher Scientific Inc., Waltham, USA). Cells were grown in EMEM (Eagle’s Minimum Essential Medium, Gibco^®^, Thermo Fisher Scientific Inc., Waltham, USA) supplemented with 10% fetal calf serum (Lonza, VWR International, Radnor, USA), 10 U of penicillin and 50 μg/mL streptomycin at 37°C and 5% CO_2_. For each set of experiments, cells were seeded at the density of 10^4^ cells/well in a 96-well culture microplate and incubated with 100 μL of the extract at concentrations ranging from 0.016 to 312 μg/mL diluted in complete medium. In addition, cells were tested with dilutions of amphotericin B (0.002 to 5 μg/mL) and DMSO (0.0015 to 3.12%). Positive controls containing cells in regular medium were also included.

Cells were grown for 24 h at 37°C, then the medium was discarded, cells were replenished with 100 μL of new complete medium and 10 μL of the ready-to-use alamarBlue reagent were added to wells. The ingredient, resazurin (no-fluorescent form), is a cell permeable compound that, upon entering cells, is reduced to resorufin (fluorescent form) as cellular innate metabolic activity results. Reduction of alamarBlue was quantitatively evaluated following a 4 and 24 h of incubation by fluorescence measurement at the Varioskan Flash Multimode Reader (Thermo Fisher Scientific Inc., Waltham, USA) using a fluorescence excitation peak at 570 nm and emission peak at 585 nm. All determinations were confirmed by replication of three experiments.

### Data analysis

**Figure 1:**
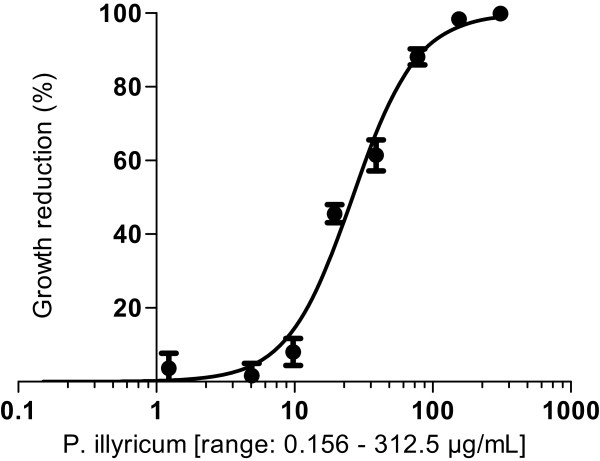
**Representative dose–response curve of a Candida albicans clinical isolate.** Experimental IC_50_ values were obtained by the interpolation of data on the specific dose–response curve, generated by means of a nonlinear regression analysis. In particular, the reduction of the yeast growth, expressed as percentage, was plotted against the drug concentration, in logarithm scale, leading to the generation of a sigmoidal curve following the equation Y = 100/(1 + 10^((LogIC50-X)*HillSlope))), where Y =50.

The antifungal activities of the *P. illyricum*, bulbs and leaves, pure compounds (lycoyine and vittatine), and amphotericin B, as reference control, were evaluated by the determination of the MIC, corresponding to the lowest concentration giving rise to an inhibition of yeast growth ≥90% of that of the solvent DMSO or drug-free controls. Moreover, two IC_50_ values were calculated for each tested reagent: an endpoint IC_50_, defined as the lowest drug concentration leading to a yeast growth reduction of the 50%, and an experimental IC_50_, obtained by the interpolation of growth data on dose–response curves (Figure 1). These curves were determined by plotting the percentages of growth inhibition, relative to the solvent or to the positive growth controls, as function of the 12 tested concentrations (in logarithm scale). Statistical analysis were carried out by nonlinear regression method using GraphPad Prism version 5.00 for Windows (GraphPad Software, San Diego California, USA).

In the anti-collagenase assay the percentage inhibition of enzyme activity was calculated by the following formula: %inhibition = [1- (Δ Abs/min _sample_/Δ Abs/min _negative control_) × 100]

IC_50_ (concentration necessary for 50% inhibition of enzyme activity) was calculated by constructing a linear regression curve. A Lineweaver-Burk (L-B) plot was built to calculate the kinetic parameters (K_m_ expressed in mM and V_max_ in nmol/s) of the enzymatic reaction without and with *P. illyricum* bulbs extract, at the IC_50_ concentration.

Cell viability was assessed on HEL 299 cell line after 4 h and 24 h of incubation with the bulbs extract of *P. illyricum* and amphotericin B. The reduction of the non-fluorescent compound into a fluorescent form of resorufin from living cells was quantitatively measured and expressed as percentage of growth, relative to solvent or positive HEL 299 controls. A growth inhibition ≥20% was considered affecting cell proliferation [20].

## Results

### *P. illyricum*extracts and pure compounds

Bulb and leaf extracts from *P. illyricum* were found to possess a slightly different alkaloid profile, as reported in Iannello et al. [13]. In particular, besides the presence of common alkaloids found in both organs of the plant, some exclusive alkaloids only present in leaves were reported. Lycorine and vittatine turned to be present in both plant organs.

### Antifungal activity

In a preliminary set of experiments, extracts from both bulbs and leaves were assayed with conventional broth microdilution susceptibility testings on reference bacteria strains (*Staphylococcus aureus* ATCC 25923, *Staphylococcus epidermidis* ATCC 12228, *Escherichia coli* ATCC 25922, and *Klebsiella pneumoniae* ATCC 9591) and on *C. albicans* ATCC 10231. Data obtained demonstrated that *P. illyricum* extracts were completely uneffective against both Gram-positive and Gram-negative bacteria, while turned rather active against *C. albicans*. Between the bulb and the leaf extracts, the former showed a higher inhibitory effect on the yeast growth compared to the latter (IC_50_ endpoint 39 and 78 μg/mL, respectively), and, as a consequence, it was subjected to further investigations. Its effect was compared to that of pure alkaloids lycorine and vittatine, and with amphotericin B. Data on their activities are summarized in Table 1.

**Table 1:** **Results of the antifungal activities of the bulbs extract and its pure alkaloids**

	Antifungal activities		
	experimental IC _50_ (μg/mL)	endpoint IC _50_ (μg/mL)	MIC (μg/mL)
*P. illyricum* bulbs	0.65 – 47	1.22 - 78	19.5 - 156
Lycorine	0.46 – 16.95	0.89 – 28.50	3.56 – 57
Vittatine	-	-	-
Amphotericin B	0.02 – 0.23	0.039 – 0.625	0.078 – 1.25

The following range concentrations were used in the microdilution broth methods: from 312 to 0.156 μg/mL for *P. illyricum* bulbs, from 114 to 0.03 μg/mL for lycorine; from 225 to 0.05 μg/mL for vittatine and from 5 to 0.002 μg/mL for amphotericin B.

**Figure 2:**
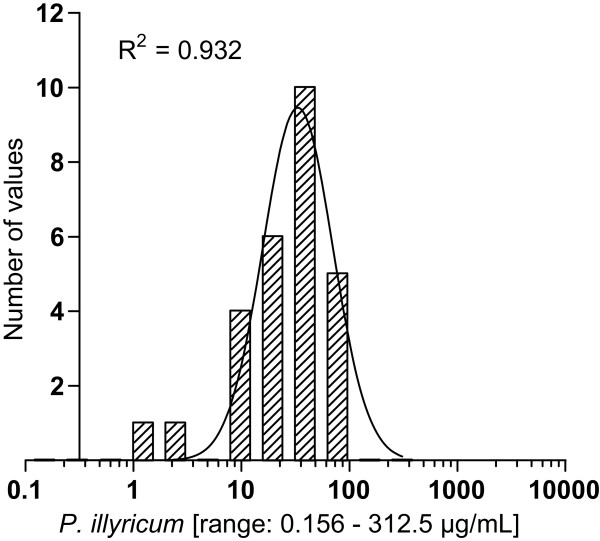
**Frequency distribution of IC**_**50**_ **values.** The number of observations were plotted as function of the endpoint IC_50_ values, in logarithm scale; data follow a Gaussian distribution (R^2^ = 0.932).

All *C. albicans* clinical isolates proved to be susceptible to the bulbs extract of *P. illyricum,* with endpoint IC_50_ values and experimental IC_50_ values ranging from 1.22 to 78, and from 0.65 to 47 μg/mL, respectively. Moreover, the frequency distribution of the endpoint IC_50_ was normally distributed (R^2^ = 0.932) with a mean value of 48.94 μg/mL and a 95% confident interval within 44.06 and 53.82 μg/mL (Figure 2). These values, significantly lower than 100 μg/mL, perfectly comply with the criteria suggested by Cos et al. [21], and demonstrate that bulbs possess a relevant and selective activity against *C. albicans* strains.

**Figure 3:**
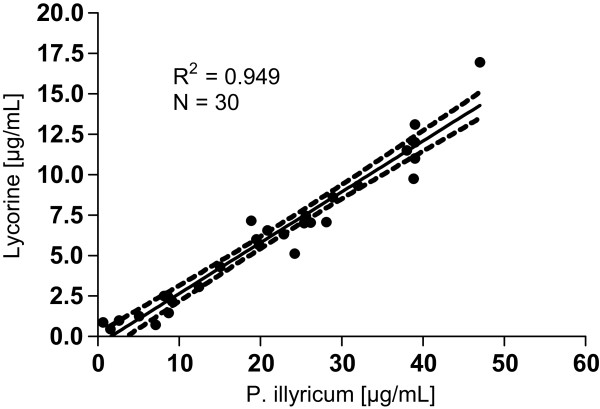
**Analysis of correlation between experimental IC**_**50**_ **values obtained for P. illyricum and lycorine on Candida albicans clinical isolates.** A scatter plot was generated with the experimental IC_50_ values obtained for *P. illyricum* bulbs and lycorine showing a positive linear pattern (R^2^ = 0.949). Dotted lines indicate the 95% confident interval.

The pure compounds, lycorine and vittatine, were assayed against the yeast isolates in order to identify the main active components in the alkaloid extract. *C. albicans* growths were not inhibited by vittatine at the tested concentrations (0.05–225 μg/mL), while lycorine potently reduced yeast growth with endpoint IC_50_ values ranging from 0.89 to 28.50 μg/mL (3.10–99.19 μM). Experimental IC_50_ values for lycorine and bulbs extract were arranged in a XY plot (Figure 3) displaying a linear correlation (R^2^ = 0.949); this result suggests that this compound is likely to contribute to the antifungal activity of the total extract of *P. illyricum.*

After IC_50_ and MIC determinations, the MFCs were measured by subculturing the supernatants from the susceptibility testings. At the tested concentrations, both *P. illyricum* extract and lycorine did not act as fungicidal agents.

All *C. albicans* isolates were susceptible to the reference amphotericin B drug with endpoint IC_50_ values and MICs ranging from 0.039 to 0.625 μg/mL and 0.078-1.25 μg/mL, respectively; MFCs were equal to MICs or two-folds higher according to the behavior of a fungicidal agent [18].

### Anti-collagenase activity

**Figure 4:**
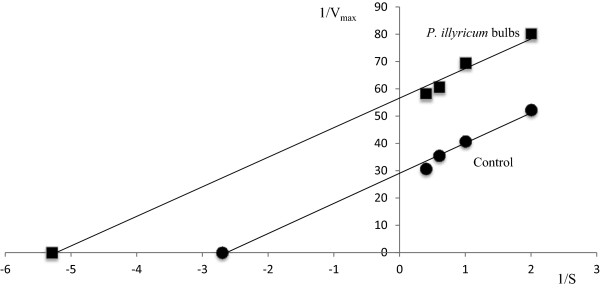
**Lineaweaver-Burk plot of Clostridium collagenase and FALGPA without (●) and with (■) P. illyricum bulb extract** ***.*** Enzyme assay was performed with a FALGPA concentration range of 0.5 – 2.5 mM.

*P. illyricum* bulb extract and pure compounds, lycorine and vittatine, were tested for their inhibiting effect on *in vitro* collagenase activity at an initial concentration of 200 μg/mL; this is the maximum concentration that can be tested with this extract without having any spectrophotometrical interference [19]. The highest anti-collagenase activity was displayed by the whole extract, reaching a 99.61% inhibitory activity, while the pure compounds, at the same concentration, showed a much lower inhibiting effect, being below 30%. The bulbs extract of *P. illyricum* was further investigated to determine IC_50_ value, which turned out to be about six times higher compared to EGCG, the reference compound for this assay (Table 2). The mechanism of action of the bulb extract towards inhibition of the collagenase enzyme has been evaluated. To this purpose, the enzyme kinetics in the absence and presence of the extract was followed, and a Lineweaver-Burk plot was built, as shown in Figure 4.

**Table 2:** **IC**_**50**_ **values of** ***P. illyricum*** **bulb extract, and of the reference compound epigallocatechin gallate**

	IC _50_ (μg/mL)	IC _50_ (μM)
*P. illyricum* bulbs	25.00 ± 0.1	-
EGCG	4.33 ± 0.02	9.45 ± 0.5

Variation of Vmax and K_M_ values in the presence of bulb extract compared to the enzyme without inhibitor suggests that the inhibition is of reversible- and uncompetitive-type. Indeed, both values turned out to be reduced by about 50% in the presence of the extract (Table 3), as a consequence of the interaction with the enzyme-substrate complex.

**Table 3:** **Kinetic parameters on the enzyme inhibition given by the extract of** ***P. illyricum***

	Enzyme with extract	Control
**K**_**M**_ **(mM)**	0.189	0.372
**V**_**max**_ **(nmol/s)**	0.017	0.033

### Cell viability testing

The analysis of cellular health of HEL 299 cell line after 24 h of incubation with the alkaloid extract of *P. illyricum* bulbs was carried out by evaluating the natural reducing power of living cells. The conversion of the substrate resazurin in resorufin by metabolically active cells was quantitatively measured at 4 and 24 h after adding the alamarBlue reagent. Both data demonstrated that the extract of *P. illyricum* did not interfere with the regular cell proliferation at the tested concentrations (data not shown).

## Discussion and conclusions

The present study was aimed at evaluating the potential of the alkaloid extract of *P. illyricum* bulbs as source for novel antimicrobial agent. While no inhibitory activity was found against bacteria, a good antifungal activity was observed on *C. albicans* clinical isolates. *In vitro* susceptibility assays were performed against 30 yeast strains concurrently with the lycorine and vittatine, alkaloids mainly present in the bulb, isolated and purified from the plant. The IC_50_ values for the bulbs, ranging from 0.65 to 47 μg/mL, demonstrate the relevant antifungal activity of *P. illyricum,* since they perfectly meet the stringent criteria for “activity” established by Cos et al. [21]. The analysis of the pure compounds allowed to identify the lycorine as the major active component on the bulbs extract. Indeed, vittatine was completely uneffective, while lycorine has a potent inhibitory effect on fungal growth, with a mean experimental IC_50_ value of 22.03 μM, again complying with the threshold (<25 μM) suggested for pure compounds [21]. In addition, the experimental IC_50_ values significantly correlate with those of the bulbs extract, corroborating that lycorine mostly contributes to the biological activities observed against *C. albicans* clinical isolates. Our results are in agreement with those obtained by other researchers [8] indicating that lycorine is not active on bacteria while is a potent inhibitor of *C. albicans* growth. The inactivity of lycorine on some bacterial strains can be explained by the fact that bacteria, as *Staphylococcus aureus*, are able to transform lycorine in its inactive metabolite 2-*O*-demethylungiminorine, instead of the active ungeremine [22]. A recent study carried out on pathogenic crop fungi suggests that lycorine may act by damaging cell membranes, leading to the exosmosis of intracellular materials [23]. Consequently, substrate absorption and cell metabolism are strongly affected.

In addition to the antifungal properties, the present study investigated the activity of *P. illyricum* against collagenase, enzyme belonging to the MMPs family [3]. Of note, *C. albicans* holds a 95 kDa metallopeptidase in the cell wall and produces extracellular proteinases, assisting the pathogen in the infectious process [24–26]. The identification of compounds with anti-metallopeptidase activity, thus targeting yeast virulence, represents a crucial step in the candidiasis drug development. Interestingly, some antibiotics, such as tetracycline, are known for their ability to inhibit the activity of some collagenases [27] than for their direct action towards microorganisms. The bulbs extract of *P. illyricum* markedly inhibited the collagenase enzyme (99.61%), with an IC_50_ value directly comparable with that of the reference standard (EGCG) while the pure compounds displayed a moderate inhibitory activity (30%), at the same enzyme concentration. In this regards, it is possible to speculate that the extract is more active than the purified compounds because, in the former, there is a synergic action of several compounds [28], and the anti-collagenase activity is not due solely to the lycorine, differently from what observed for the antifungal properties. The analysis of the mechanism of inhibition of bulbs extract of *P. illyricum* towards the collagenase enzyme reveals that the inhibition is of reversible- and uncompetitive-type, thus suggesting that an interaction with the enzyme-substrate complex occurs, bringing to a higher affinity of the enzyme towards the substrate. Thus, taken together, results obtained in the present study suggest that alkaloid extract of *P. illyricum* is a very attractive and auspicious candidate for antifungal drug development since it combines two modes of action: as conventional antimicrobial agents, the extract selectively interferes with yeast cell viability by inhibiting *C. albicans* growth (-static action) and, as an added value, it exhibits a marked anti-collagenase activity which may target fungal virulence in the tissue invasion process.

## Electronic supplementary material

Additional file 1: **GC-MS chromatogram (TIC on Y-axis) of alkaloid extracts in bulbs of** ***Pancratium illyricum*** **L. Conditions as reported in the text.** (A):_galanthamine, (B): sanguinine, (C): vittatine, (D): habranthine, (E): lycorine, (F): leucotamine, (G): O-methylleucotamine, (H): 2-hydroxyhomolycorine. (PNG 18 KB)

Additional file 2: **GC-MS of galanthamine in bulb extract of Pancratium illyricum L. and relative spectrum from library.** (ZIP 49 KB)

Additional file 3: **GC-MS of sanguinine in bulb extract of Pancratium illyricum L. and relative spectrum from library.** (ZIP 52 KB)

Additional file 4: **GC-MS of vittatine in bulb extract of Pancratium illyricum L. and relative spectrum from library.** (ZIP 56 KB)

Additional file 5: **GC-MS of habranthine in bulb extract of Pancratium illyricum L. and relative spectrum from library.** (ZIP 47 KB)

Additional file 6: **GC-MS of lycorine in bulb extract of Pancratium illyricum L. and relative spectrum from library.** (ZIP 45 KB)

Additional file 7: **GC-MS of 2-hydroxyhomolicorine in bulb extract of Pancratium illyricum L. and relative spectrum from library.** (ZIP 25 KB)
