# Supplementary figures and images for: Relevant and selective activity of Pancratium illyricum L. against Candida albicans clinical isolates: a combined effect on yeast growth and virulence
Source: BMC Complement Altern Med. 2014 Oct 23;14:409. doi: 10.1186/1472-6882-14-409 (PMC4213485; doi:10.1186/1472-6882-14-409)

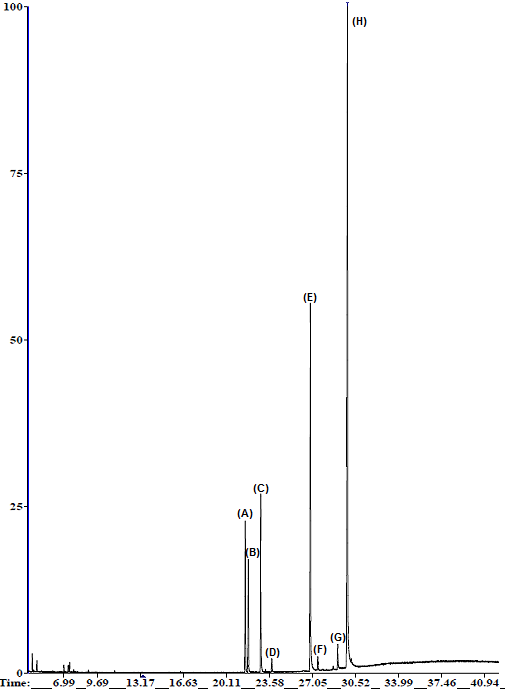

Supplement: Supplementary file 1 — Additional file 1: GC-MS chromatogram (TIC on Y-axis) of alkaloid extracts in bulbs of Pancratium illyricum L. Conditions as reported in the text. (A):_galanthamine, (B): sanguinine, (C): vittatine, (D): habranthine, (E): lycorine, (F): leucotamine, (G): O-methylleucotamine, (H): 2-hydroxyhomolycorine. (PNG 18 KB) [file 12906_2014_1984_MOESM1_ESM.png]

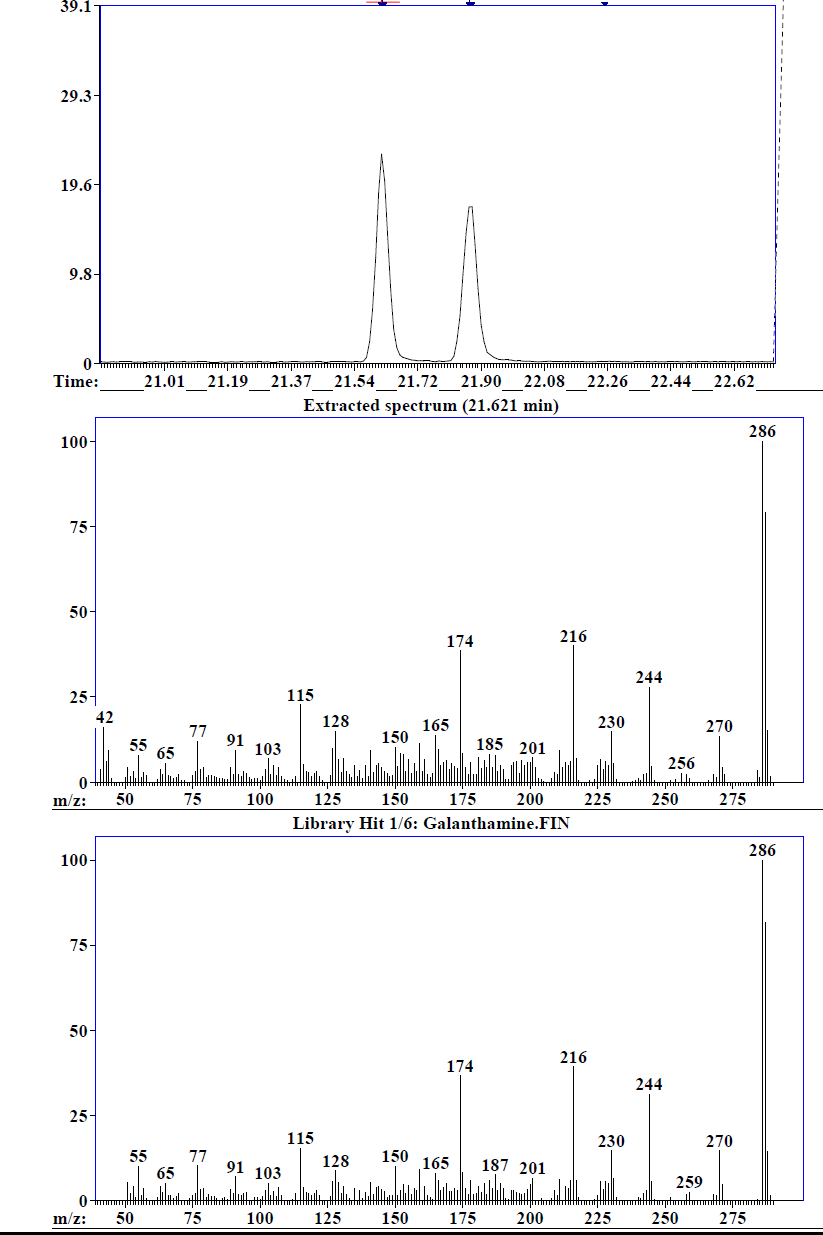

Supplement: Supplementary file 2 — Additional file 2: GC-MS of galanthamine in bulb extract of Pancratium illyricum L. and relative spectrum from library. (ZIP 49 KB) [file 12906_2014_1984_MOESM2_ESM.zip › 6853893401340299_MOESM2_ESM.bmp]

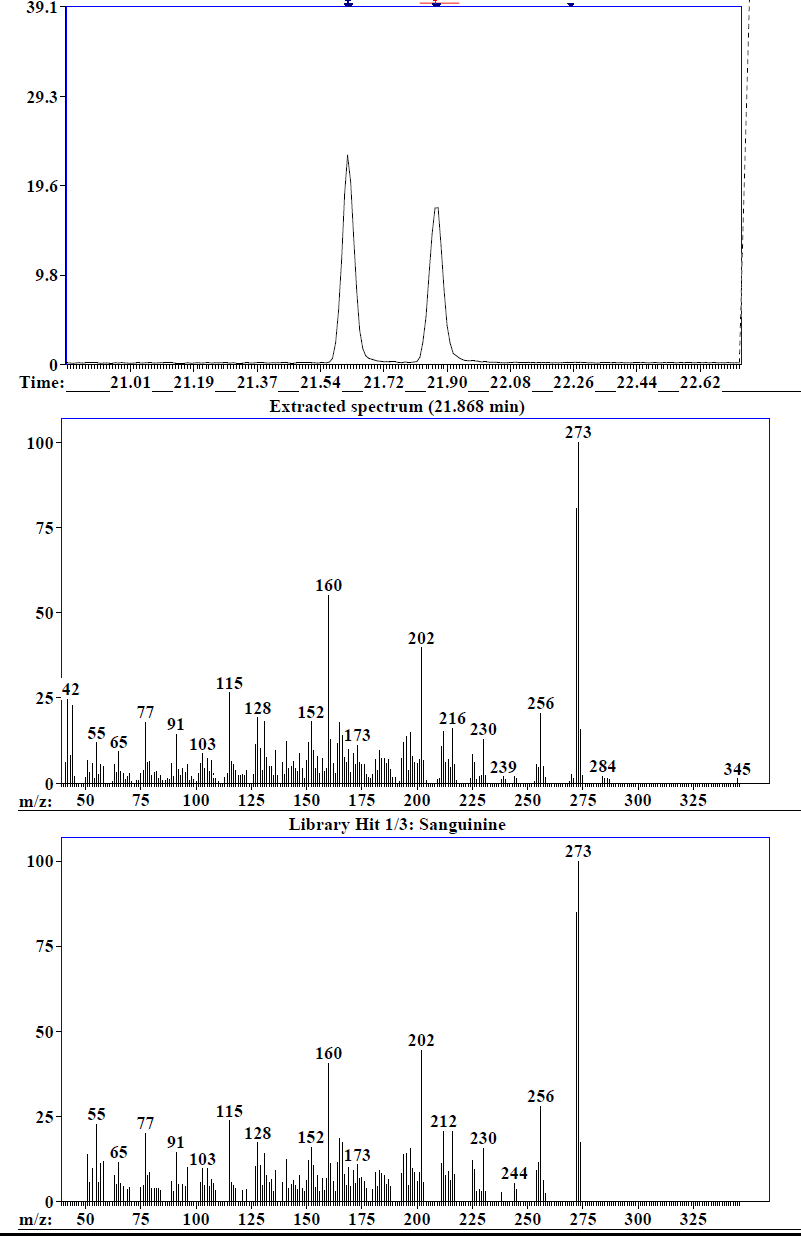

Supplement: Supplementary file 3 — Additional file 3: GC-MS of sanguinine in bulb extract of Pancratium illyricum L. and relative spectrum from library. (ZIP 52 KB) [file 12906_2014_1984_MOESM3_ESM.zip › 6853893401340299_MOESM3_ESM.bmp]

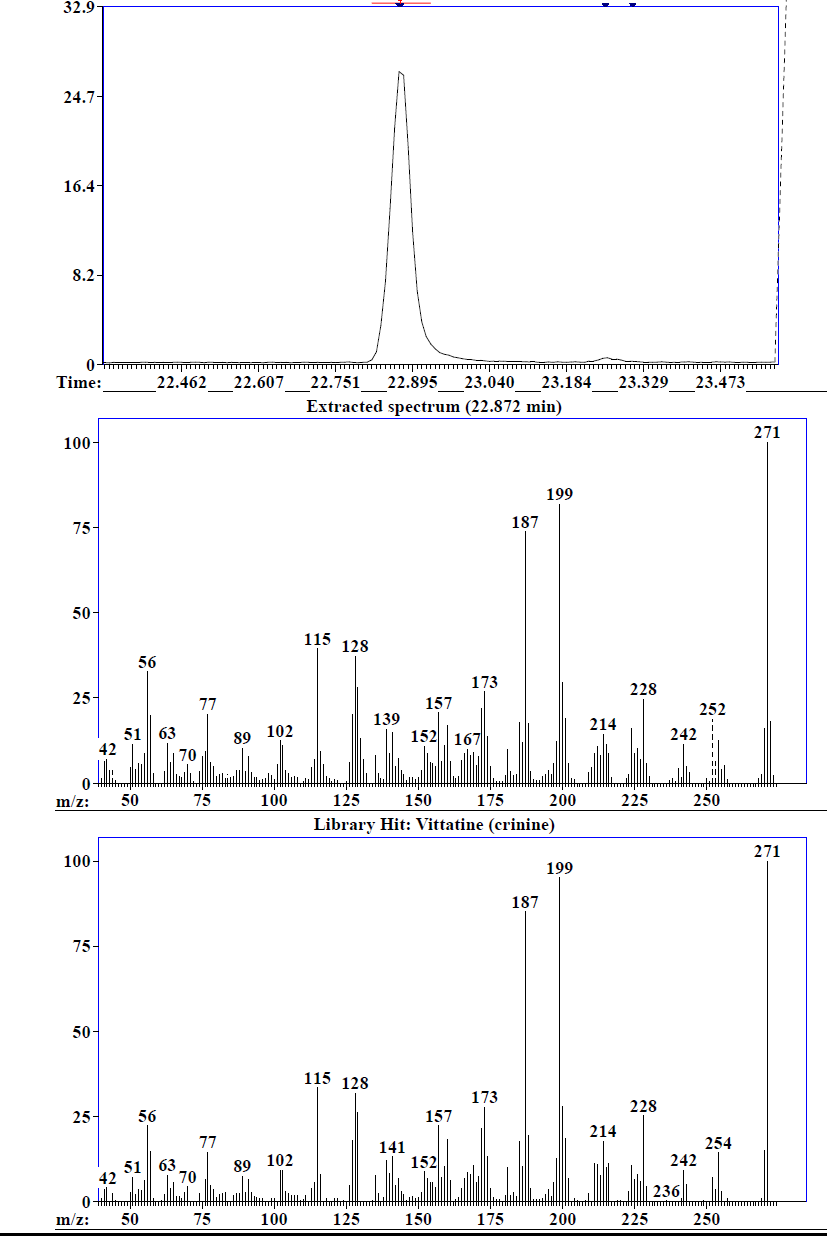

Supplement: Supplementary file 4 — Additional file 4: GC-MS of vittatine in bulb extract of Pancratium illyricum L. and relative spectrum from library. (ZIP 56 KB) [file 12906_2014_1984_MOESM4_ESM.zip › 6853893401340299_MOESM4_ESM.bmp]

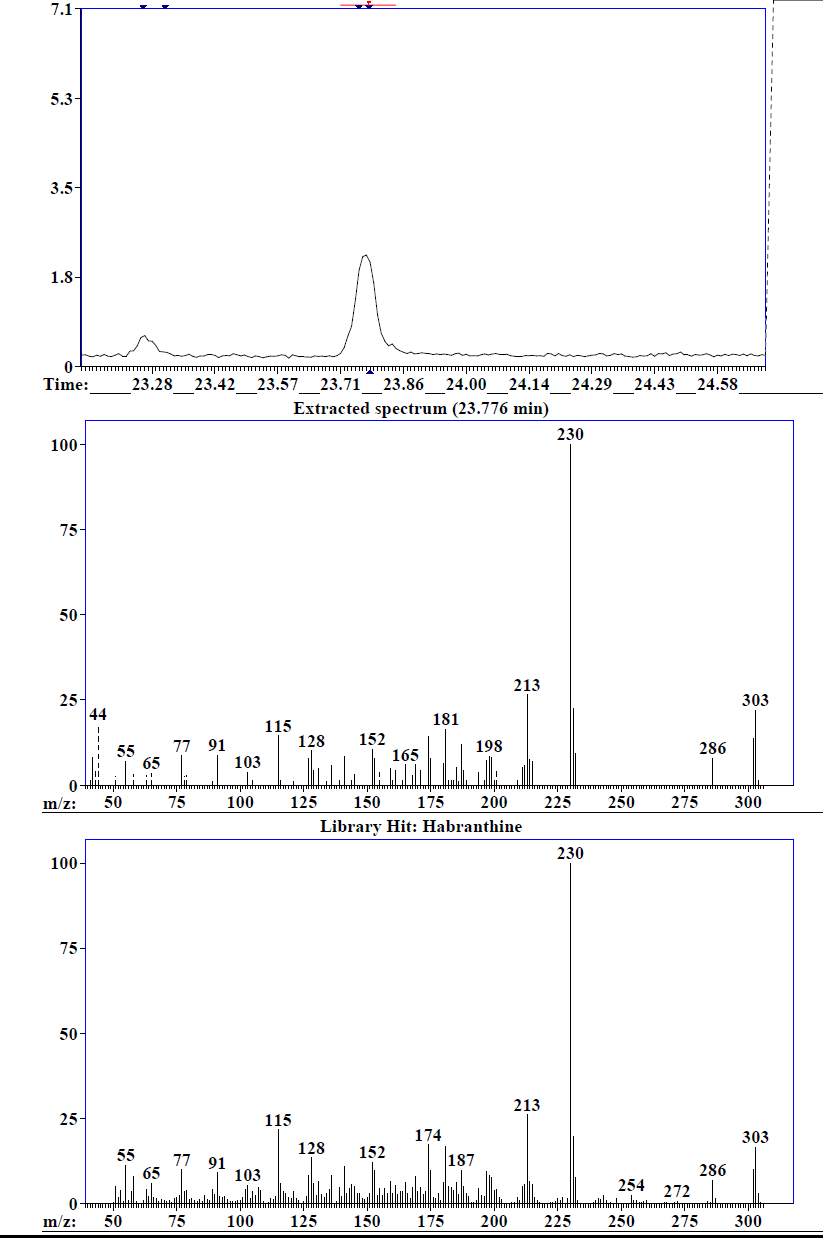

Supplement: Supplementary file 5 — Additional file 5: GC-MS of habranthine in bulb extract of Pancratium illyricum L. and relative spectrum from library. (ZIP 47 KB) [file 12906_2014_1984_MOESM5_ESM.zip › 6853893401340299_MOESM5_ESM.bmp]

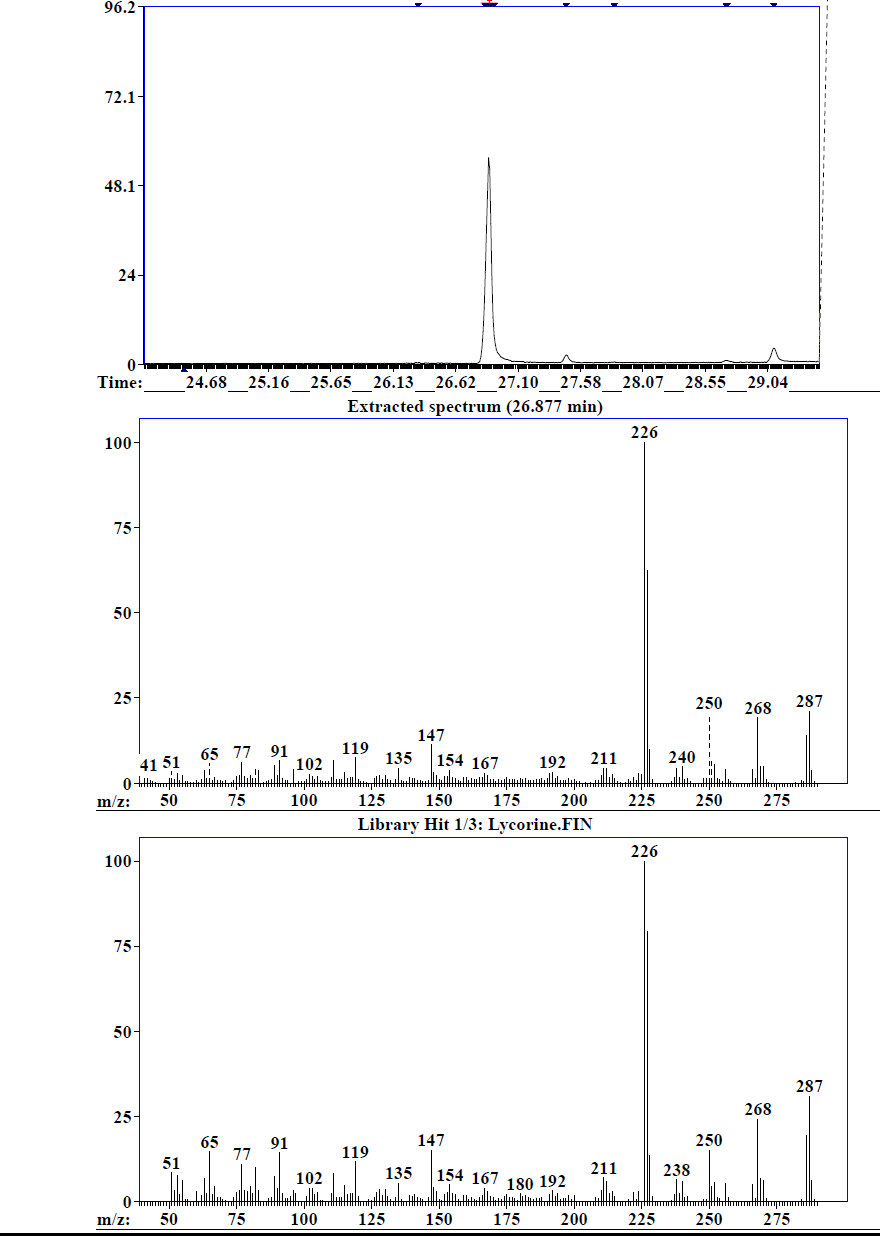

Supplement: Supplementary file 6 — Additional file 6: GC-MS of lycorine in bulb extract of Pancratium illyricum L. and relative spectrum from library. (ZIP 45 KB) [file 12906_2014_1984_MOESM6_ESM.zip › 6853893401340299_MOESM6_ESM.bmp]

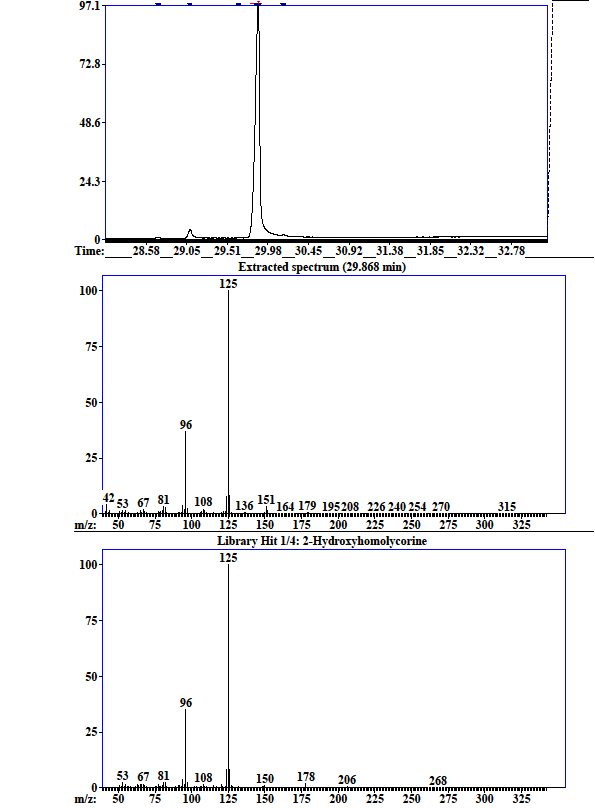

Supplement: Supplementary file 7 — Additional file 7: GC-MS of 2-hydroxyhomolicorine in bulb extract of Pancratium illyricum L. and relative spectrum from library. (ZIP 25 KB) [file 12906_2014_1984_MOESM7_ESM.zip › 6853893401340299_MOESM7_ESM.bmp]
